# Serotonin-Exacerbated DSS-Induced Colitis Is Associated with Increase in MMP-3 and MMP-9 Expression in the Mouse Colon

**DOI:** 10.1155/2016/5359768

**Published:** 2016-07-05

**Authors:** Menglu Chen, Lei Gao, Pan Chen, Dandan Feng, Yalin Jiang, Yongchao Chang, Jianjun Jin, Fong-Fong Chu, Qiang Gao

**Affiliations:** ^1^Department of Gastroenterology and Hepatology, The First Affiliated Hospital and College of Clinical Medicine, Henan University of Science and Technology, Luoyang 471003, China; ^2^Department of Clinical Laboratory, The First Affiliated Hospital and College of Clinical Medicine, Henan University of Science and Technology, Luoyang 471003, China; ^3^Department of Cancer Genetics and Epigenetics, Beckman Research Institute of City of Hope, Duarte, CA 91010, USA; ^4^Department of Gastroenterology and Hepatology, Beijing Rehabilitation Hospital of Capital Medical University, 1 Gongliao Road, Shijingshan District, Beijing 100114, China

## Abstract

*Background*. 5-HT enhances dextran sulfate sodium- (DSS-) induced colitis and is involved in inflammatory bowel disease (IBD). Matrix metalloproteinases (MMPs) play roles in the process of intestinal inflammation.* Aims*. To examine whether 5-HT induces MMPs expression in mouse colon to enhance DSS-induced colitis.* Materials and Methods*. C57BL/6J (B6) mice were treated with either low-dose (1.0 mg/kg) or high-dose (2.0 mg/kg) 5-HT by enema, low-dose (1.0%) or high-dose (2.5%) DSS, or combined low-dose (1.0%) DSS and (1.0 mg/kg) 5-HT. Mouse colitis was analyzed. MMPs and tissue inhibitors of MMPs (TIMPs) mRNA were measured by real-time quantitative RT-PCR in mouse colon and in human Caco-2 cells and neutrophils. MMP-3 and MMP-9 protein levels were quantified from immunohistochemistry (IHC) images of mouse colons.* Results*. 5-HT exacerbated DSS-induced colitis, low-dose 5-HT induces both MMP-3 and MMP-9, and high-dose 5-HT only increased MMP-3 mRNA expression in mouse colon. Mouse colon MMP-3 and MMP-9 protein levels were also elevated by 5-HT treatment. The MMP-2, TIMP-1, and TIMP-2 mRNA levels were increased in the inflamed colon. 5-HT induced MMP-3 and MMP-9 mRNA expression in Caco-2 and human neutrophils, respectively, in vitro.* Conclusion*. 5-HT induced MMP-3 and MMP-9 expression in mouse colon; these elevated MMPs may contribute to DSS-induced colitis.

## 1. Introduction

The inflammatory bowel disease (IBD), comprised of ulcerative colitis (UC) and Crohn's disease (CD), is the recurrent chronic idiopathic inflammatory disease of the GI tract [1–3]. Although the precise etiology of IBD remains unknown, a variety of inflammatory mediators and enzymes, such as cytokines, growth factors, reactive oxygen species (ROS), GI hormones, and matrix metalloproteinases (MMPs), are known to regulate inflammation and are involved in pathogenesis of IBD [4, 5].

5-Hydroxytryptamine (5-HT, serotonin), a monoamine neurotransmitter, is biochemically derived from tryptophan. Tryptophan hydroxylase (TPH) is a rate-limiting enzyme in the synthesis of 5-HT and is located prominently in enterochromaffin (EC) cells [6]. Approximately 90% of 5-HT in humans is synthesized in gastrointestinal (GI) EC cells by tryptophan hydroxylase-1 (TPH1) [7], and 5% of 5-HT is synthesized in myenteric neurons [8] and brain by TPH2 [9]. 5-HT is an important mucosal regulatory molecule, which mediates gut motility and secretary functions, promotes growth and turnover of mucosal epithelium, and maintains intestinal homeostasis [10]. 5-HT is also a neuron transmitter in the central nervous system to regulate mood and cause vasoconstriction [10, 11]. In the intestinal mucosa, the serotonin reuptake transporter (SERT) takes up 5-HT into epithelial cells and serotonergic neurons and inactivates it [12]. 5-HT exerts its functions by binding to 5-HT receptors which are widely expressed in intestinal tissues [13, 14]. 5-HT is involved in the pathogenesis of intestinal disorders, including IBD [15–17] and irritable bowel syndrome [15, 18, 19]. The immunomodulatory effects of 5-HT are associated with gut inflammation [11]. The changes in EC cell numbers and 5-HT contents in colon mucosa of IBD patients have been reported [15, 20]. Mice treated with dextran sulfate sodium (DSS) to induce colitis also have increased 5-HT levels contributed by higher number of EC cells and decreased SERT mRNA expression [21]. 5-HT also exacerbated trinitrobenzene sulfonic acid- (TNBS-) induced mouse colitis and is believed to play a key role in pathogenesis of experimental colitis [22, 23].

MMPs are a class of structurally related zinc- and calcium-dependent enzymes, which are responsible for the metabolism of extracellular matrix (ECM) via the remodeling degradation of most components of the ECM [24, 25]. To date, 23 distinct MMPs have been identified in humans (24 in mice) [26]. MMPs facilitate cell migration and cell infiltration through degradation of basement membrane and ECM [24, 27]. MMPs catalyze proteolytic cleavage of biologically active protein molecules, such as cytokines, growth factors, and chemokines, from a membrane-anchored inactive form into a free active form to maintain tissue homeostasis. Under physiological conditions, MMPs are secreted as a latent form of enzyme at low levels and are conversed to active enzymes by proteolytic cleavage [28]. The MMP proteins are tightly controlled at multiple levels, including transcription, translation, secretion, and activation [25, 26]. Additionally, MMP proteins are also regulated by tissue inhibitors of MMPs (TIMPs), which are endogenous inhibitors of MMPs. TIMPs consist of four members, TIMP-1, TIMP-2, TIMP-3, and TIMP-4, all of which regulate MMP activities by forming a 1 : 1 complex with the high zinc binding site of MMPs. TIMPs are important regulators of tissue remodeling and cellular behavior [29, 30].

In active IBD, many MMPs and TIMPs, including MMP-2, MMP-3, and MMP-9 and TIMP-1 and TIMP-2 were elevated in the flamed tissues compared to the noninflamed tissue [31–33]. MMP-2 (gelatinase A) and MMP-9 (gelatinase B) are able to proteolytically degrade gelatin. Under physiological condition MMP-2 is ubiquitously expressed [26]. MMP-2 appears to be protective against DSS-, TNBS-, and* Salmonella typhimurium*-induced colitis [34]. MMP-9 is mainly secreted from inflammatory cells, such as neutrophils, monocytes, and macrophages and specially degrades basement membrane type IV collagen, as well as other ECM components [27, 35]. Of the MMPs, MMP-9 is the most abundantly expressed in inflamed tissues of IBD patients [33]. MMP-3 (stromelysin-1) is secreted by epithelial cells, fibroblasts, vascular smooth cells, endothelial cells, and macrophages [26, 36–39]. MMP-3 degrades ECM components such as fibronectin, denatured collagens (gelatin), laminin, and proteoglycans [26]. Both MMP-3 and MMP-9 are believed to be involved in the process of IBD pathology [40, 41]. Proinflammatory cytokines, such as IL-6, IL-8, and TNF-*α*, can induce the transcription, translation, and secretion of MMPs [32, 42, 43].

DSS-induced colitis is an animal colitis model resembling ulcerative colitis. Although the mechanism by which DSS induces colitis is not clear, DSS damaged colon epithelium is highly relevant to human colitis [44]. It has been shown that DSS increases MMP-2, MMP-3, and MMP-9 mRNA levels in colon [45, 46]. In this study, we explored whether 5-HT exacerbated DSS-induced colitis can be contributed by MMPs expression in the colon.

## 2. Material and Methods

### 2.1. Animal Studies

C57BL/6J (B6) mice purchased from Vital River Laboratory Animal Technology Co. Ltd. (Beijing, China) were maintained in ventilated cages with free access to food and water. Mice were fed with rodent chow from Beijing HFK Bioscience Co. Ltd. (Beijing, China). Animal Care and Use Committee in the hospital approved all procedures in this study.

Male mice, at 6–8 weeks of age, with body weight of 20–25 g, were divided into 6 groups treated with 5-HT (MW 212.68, Sigma, St Louis, USA, Cat. number H9523) or DSS (MW 36–50 kDa, MP Biomedicals, Santa Ana, USA, Cat. number 160110). These are (i) low-dose (1.0 mg/kg) 5-HT (*n* = 9) and (ii) high-dose (2.0 mg/kg) 5-HT (*n* = 9), both groups receiving 5-HT in saline by enema at days 1 and 4 [22]; (iii) low-dose (1%) DSS (*n* = 14) and (iv) high-dose (2.5%) DSS (*n* = 7), both groups receiving DSS in drinking water for 5 days; (v) DSS + 5-HT (*n* = 12), mice being treated with 1.0% DSS and 1 mg/kg 5-HT concurrently; (vi) control group (*n* = 8), mice receiving enema with saline. Mice were monitored daily for body weight and signs of colitis, including diarrhea and perianal ulceration to obtain disease activity index (DAI). Mice were euthanized at day six.

After euthanasia, the colon length was measured, and the sera were collected. The colon tissue was washed in PBS, and then half was fixed in 10% formalin and embedded in paraffin. Sections were stained by hematoxylin and eosin (H&E) for pathological examination or by immunohistochemistry (IHC). The other half of colon was stored at −80°C for RNA extraction. RNA*later* (Qiagen, Hilden, Germany) was used immediately when the samples were collected to prevent RNA degradation. The severity of colitis was evaluated by microscopic examination of colon tissues. Each mouse was scored for inflammation and pathology in a blinded fashion using a modified system as we described previously [47]. These include lymphocytes and neutrophil infiltration (0–3 points), Paneth cell and goblet cell degranulation (0–2 points), epithelium reactivity such as crypt distortion (0–3 points), and inflammatory foci (0–3 points). The threshold for detection of severe acute inflammation corresponds to scores of 6 in this study.

### 2.2. Immunohistochemical (IHC) Staining

Four-micron sections of paraffin-embedded samples were mounted on poly-L-lysine-coated slides. IHC was performed using a modified biotin-peroxidase complex method as described previously [31]. Sections were then incubated overnight with a rabbit polyclonal antibody for MMP-3 (1 : 100, Abcam, London, England, Cat. number ab52915). The antigen-antibody complex was detected with biotinylated goat anti-rabbit antibody (1 : 300, ZSBio com, Beijing, China, Cat. number PV-9000) after hybridized with streptavidin-HRP, which was visualized by reacting with 3,3-diaminobenzidine. MMP-9 was detected by a goat polyclonal antibody (1 : 100 dilution, Santa Cruz, CA, USA, Cat. number sc-6840) as primary antibody and secondary antibody was a biotinylated rabbit anti-goat antibody (1 : 300 dilution, Boster Biological Technology Co. Ltd, Wuhan, China, Cat. number SA1023). The sections were counterstained with hematoxylin. The negative control sections were obtained by omitting the primary antibody or using an unrelated rabbit polyclonal antibody.

The MMP-3 and MMP-9 protein levels were evaluated by a blind way in 10 fields under 400x magnification from each slide. One hundred cells per field were categorized as follows: “−”, 0%, no staining; “+”, >25% of cells were stained; “++”, 26 to 50% of cells were stained; and “+++”, >50% of cells were stained.

### 2.3. Quantitative Real-Time PCR (qPCR)

qPCR was performed as we described previously [31]. Briefly, total RNA was extracted using TRIzol Reagent (Invitrogen, USA) according to the manufacturer's instruction. The cDNA was synthesized using PrimeScript*™* RT Master Mix (Takara, Japan). The primer sequences for human and mouse samples (Table 1) were designed with Primer3.0 [48] and the primers were synthesized by Sangon Biotechnology (Zhengzhou, China). The reaction mixture of real-time qPCR was performed using a CFX96*™* Real-Time PCR system (BIO-RAD, USA), and the cyber green was used to detect PCR products. When the primers annealing temperature was 60°C or more, a two-step PCR reaction method was used. When the primers annealing temperature was below 60°C, a three-step method was used. Each sample was assayed in triplicate. The efficiency of PCR amplification was 97% to 105%. A melting curve analysis was performed for the PCR products of each target gene and *β*-actin to evaluate primer specificity. The relative abundance of target gene mRNA level was evaluated using the comparative Ct (2^−ΔΔCt^) method and was normalized to *β*-actin mRNA level. The mRNA expression level was presented by the relative fold by comparing the quantity of mRNA in different groups. When the Ct value is higher than 35, the gene is considered as unexpressed.

### 2.4. Cell Culture

Caco-2, a human colon enterocyte-like cell line (ATCC), was cultured at 37°C in an atmosphere of 5% CO_2_ and maintained in high-glucose DMEM supplemented with 2 mM glutamine, 100 U/mL penicillin, 100 *μ*g/mL streptomycin, 1% nonessential amino acids, and 10% heat-inactivated fetal bovine serum (FBS) (Hyclone, Utah, USA). For 5-HT stimulation experiments, cells were seeded in 12-well plates at a density of 1.25 × 10^4^ cells/well. The cells were serum-starved for 24 h before adding 5-HT to minimize the effect of 5-HT present in the FBS. 5-HT was added to the serum-free medium at different concentrations for up to 24 h.

Heparinized blood samples were collected from consented healthy volunteers. The blood samples were seeded in 6-well plates at a volume of 1.5 mL/well of DMEM and were stimulated with 10 *μ*M 5-HT for 24 h. Leucocytes were isolated from the wells by the addition of erythrocyte lysis buffer, containing 0.16 M NH_4_Cl, 10 mM KHCO_3_, and 0.01 mM K_2_-EDTA (pH 7.4) at 4°C. The lysate was transferred into a 15 mL centrifuge tube and centrifuged at 1500 rpm, 4°C for 5 min to pellet leucocytes, which were immediately processed for RNA isolation and determination of MMP-9 mRNA levels.

### 2.5. Statistical Analysis

Student's *t*-test, one-way analysis of variance (ANOVA), and Mann-Whitney *U* test were used to compare the data from different groups. The significant difference was defined as *P* < 0.05. Data was reported as means ± standard deviations (SD). All statistical analysis was performed by using the SPSS 19.0 statistics package (SPSS Inc., Chicago, IL, USA).

## 3. Results

### 3.1. 5-HT Exaggerated DSS-Induced Colitis

**Figure 1:**
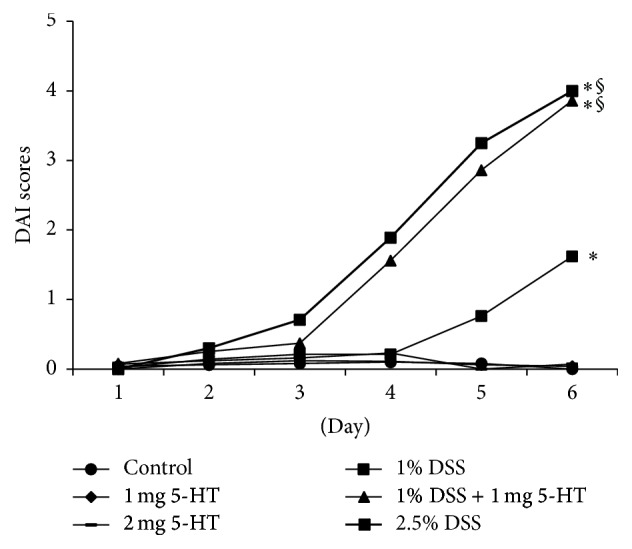
DAI scores of mice treated with 5-HT or/and DSS. DAI scores were analyzed in six groups of mice. The control group (*n* = 8) and those receiving 1.0 mg/kg (*n* = 9) or 2.0 mg/kg 5-HT (*n* = 9) had low levels of DAI. The group treated with 1.0% DSS (*n* = 14) had elevated DAI scores. Groups receiving 1.0% DSS plus 1.0 mg/kg 5-HT (*n* = 12) and 2.5% DSS (*n* = 7) had further increased DAI scores. Multiple comparisons among groups were done. ^*∗*^ *P* < 0.05 versus mice in control, 1.0 mg/kg, or 2.0 mg/kg group. ^§^ *P* < 0.05 versus mice 1% DSS group.

**Figure 2:**
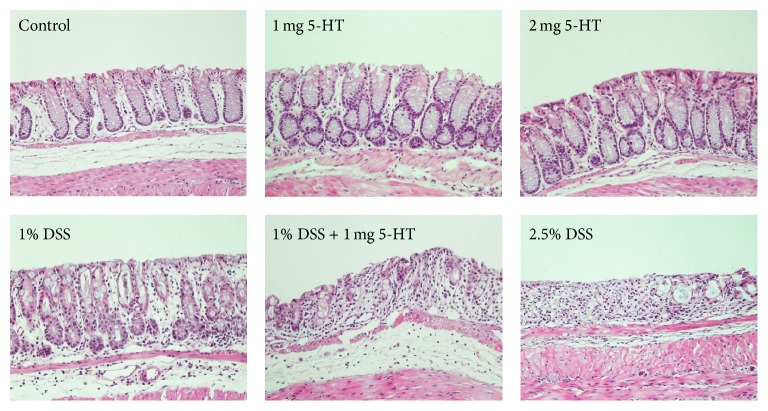
Histopathological analysis of mouse colon treated with 5-HT or/and DSS. There was no colitis in control group (*n* = 8) or alone receiving 1.0 mg/kg (*n* = 9) or 2.0 mg/kg 5-HT (*n* = 9). Mild colitis was in mice treated with 1.0% DSS (*n* = 14). Both mice receiving 1.0% DSS plus 1.0 mg/kg 5-HT (*n* = 12) and 2.5% DSS (*n* = 7) had significant severe colitis, demonstrating the increased number of infiltrating inflammatory cells, crypt loss, and erosion of the mucosa and submucosa.

Similar to Regmi's study that 5-HT exaggerated TNBS-induced colitis, we found 5-HT also exacerbated DSS-induced colitis [22] (Table 2, Figures 1 and 2). 5-HT alone, at either low (1.0 mg/kg) or high (2.0 mg/kg) dosage, did not induce colitis determined by DAI and histopathological analysis. Mice treated with 1% DSS had signs of mild colitis, including diarrhea and loss of body weight shown at day 5, but the weight loss was less than 10% at day 6. Mild inflammation was seen in colon tissue sections, including increased infiltration of inflammatory cells in the mucosa and submucosa, mild crypt distortion, and shortened crypt depth. Mice drinking 2.5% DSS suffered severe colitis, diarrhea, bloody stools, and losing 21% of body weight at day six. Mice receiving both 1% DSS and 1 mg/kg 5-HT had a similar extent of colitis as those receiving high-dose DSS. The shortened colon was associated with the increased DAI. The pathological analysis demonstrated severe inflammation, including ulcerative lesions, increased number of infiltrating inflammatory cells, crypt loss, and erosion of the mucosa and submucosa.

While 5-HT alone did not induce colitis, high-dose 5-HT significantly increased the levels of proinflammatory cytokine IL-6 and chemokine IL-8 mRNA in the colons (Table 3). DSS alone strongly induced IL-6 mRNA and TNF-*α*, but not IL-8 mRNA levels. Combined 1% DSS and 1 mg/kg 5-HT treatment produced the same pattern of cytokine/chemokine expression as the DSS treatment, that is, no induction of IL-8 mRNA levels.

### 3.2. 5-HT Increased MMP-3 and MMP-9, but Not MMP-2, TIMP-1, and TIMP-2, mRNA Levels in Mouse Colon

**Figure 3:**
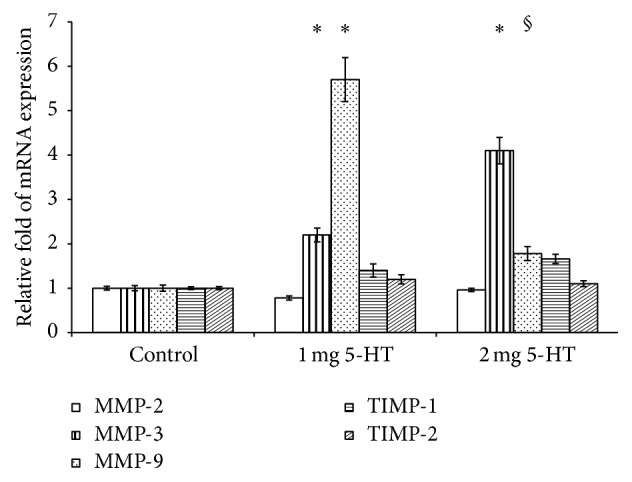
5-HT alone increased MMP-3 and MMP-9 mRNA expressions in mouse colon. The treatment with low-dose 5-HT increased MMP-9 mRNA expression in mouse colon tissues; high dose only increased MMP-3 mRNA. Neither low- nor high-dose 5-HT increased the expression of MMP-2, TIMP-1, or TIMP-2 mRNA. Results are expressed as the mean ± standard deviations (SD) and 8 mice were included for all three groups. Multiple comparisons among group were done. ^*∗*^ *P* < 0.05 versus control mice. ^§^ *P* < 0.05 versus 1 mg 5-HT group.

MMPs play an important role in tissue modeling and damage in IBD [31, 41], and the proteolytic activity of MMPs is tightly regulated by their natural inhibitors, TIMPs [29, 30]; we explored whether MMP-2, MMP-3, MMP-9, TIMP-1, and TIMP-2 mRNA expressions are induced by 5-HT in mouse colons. We found that 5-HT alone increased both MMP-3 and MMP-9 mRNA levels; low-dose and high-dose 5-HT increased MMP-3 mRNA by 2- and 4-fold, respectively (Figure 3). Low-dose 5-HT also induced MMP-9 mRNA by 5.5-fold, although high-dose 5-HT did not increase MMP-9 mRNA levels significantly. Contrary to MMP-3 and MMP-9 mRNA expressions, 5-HT alone did not affect MMP-2, TIMP-1, and TIMP-2 mRNA expression.

Consistent with the literature, DSS-treated colons had elevated expression of MMP-2, MMP-3, MMP-9, TIMP-1, and TIMP-2 mRNAs with the exception of TIMP-2 mRNA in low-dose DSS-treated colon (Table 4). Induction of MMP-2 and TIMP-2 by DSS and DSS + 5-HT was rather modest; MMP-2 was increased 1.8- to 6.3-fold and TIMP-2 was increased 3.8- to 6.0-fold. Induction of MMP-3, MMP-9, and TIMP-1 reached 2-log higher in mice treated with either DSS + 5-HT or high-dose DSS compared with the control mice.

### 3.3. 5-HT Increased MMP-3 and MMP-9 Protein Levels in Mouse Colon Analyzed by IHC

**Figure 4:**
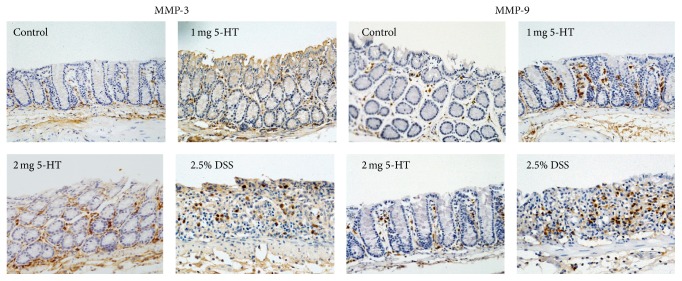
IHC staining of MMP-3 and MMP-9 in mouse colon tissue. The low expression MMP-3 was in colon tissue of control mice. The expression of MMP-3 was increased in the colon tissue of mice treated with high-dose 5-HT, further increased in severe inflammatory tissue. MMP-3 mainly located in infiltrating cells and stromal cells, some epithelial cell having weak staining. MMP-9 was increased in low 5-HT group but not in high-dose group and also highly increased in tissue with severe inflammation. MMP-9 was located mainly in inflammatory cells. Six mice from each group were included for IHC staining.

We performed MMP-3 IHC and found that MMP-3 was located at epithelial cells, endothelial cells, and some infiltrating cells in the lamina propria (Figure 4, top panels). We quantified the protein levels based on the number of positively stained cells in the field. Consistent with gene expression, the MMP-3 protein levels were elevated 3.7-fold in the colons of mice treated with high-dose 5-HT, although the smaller increase of MMP-3 protein in mice treated with low-dose 5-HT is not significantly higher than the control mice (Table 5).

MMP-9 was expressed mainly in the infiltrating inflammatory cells (Figure 4, lower panels). Similar to the mRNA expression pattern, MMP-9 protein was elevated in the colons of mice treated with low-dose, but not high-dose, 5-HT. As expected, the colons of DSS-treated mice had nearly 10-fold higher MMP-3 and MMP-9 proteins than the control colons (Table 5).

### 3.4. 5-HT Increases MMP-3 in Caco-2 Cells and MMP-9 mRNA in Leucocytes

**Figure 5:**
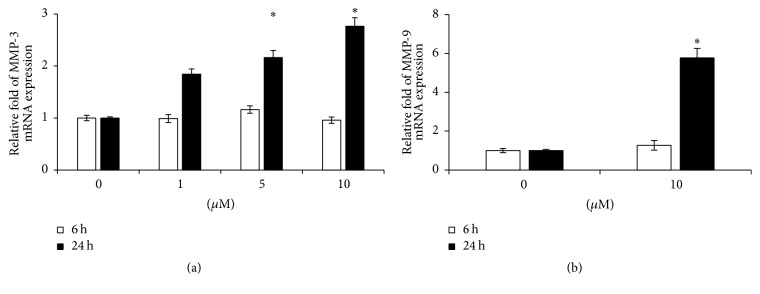
The expression of MMP-3 and MMP-9 mRNA in Caco-2 cells and human leucocytes. After 24 h stimulation with 5-HT for 24 h, MMP-3 mRNA expression in Caco-2 cells was significantly increased at the concentration of 5 and 10 *μ*M (a). The expression of MMP-9 mRNA was up to 6-fold increase compared to the leucocytes without 5-TH stimulation (b). The data represent 4 experiments. ^*∗*^ *P* < 0.05 versus cells without treatment with 5-HT.

Since MMP-3 is expression in colon epithelial cells and MMP-9 is mainly synthesized in leucocytes [26, 35], we tested the direct effect of 5-HT on the expression MMP-3 in Caco-2 cells and MMP-9 mRNA in leucocytes. In Caco-2 cells, 5-HT significantly increased the MMP-3 mRNA levels after 24 h treatment with either 5 or 10 *μ*M 5-HT compared to the control cells (Figure 5(a)). MMP-9 mRNA level was not changed under the same conditions (data not shown). MMP-9 mRNA level in leucocytes was also increased 6-fold after 24 h treatment with 10 *μ*M 5-HT (Figure 5(b)). No MMP-3 mRNA was detected in leucocytes (data not shown).

## 4. Discussion

5-HT is involved in pathogenesis of IBD [15–17] and is well documented to exacerbate chemical-induced colitis in mice [22, 23]. The development of IBD is associated with changes of EC cells [49, 50]. The elevated EC cell number and decreased SERT mRNA expression lead to increase in 5-HT content, which is associated with the pathogenesis of DSS-induced colitis [21]. Since MMPs are upregulated in IBD and other inflammatory conditions and DSS treatment induced MMP-2, MMP-3, and MMP-9 mRNA levels in mouse colon [45, 46], we analyzed the direct effect of 5-HT on the expression of these MMP genes. We found 5-HT alone induced MMP-3 and MMP-9 but not MMP-2 gene expression in the colon. Furthermore, we showed the direct effect of 5-HT induction of MMP-3 expression in Caco-2 cells and MMP-9 expression in leucocytes. Thus, we provided the first evidence to show that 5-HT has a direct effect on the induction of MMPs in two different cell types.

5-HT-induced MMP-3 and MMP-9 gene expression may be mediated by induction of proinflammatory cytokines, since IL-6 and IL-8 mRNA levels are significantly elevated in the colons of mice treated with high-dose 5-HT. Induction of the same proinflammatory cytokines is in rat colons after being treated by 1.0 mg/kg 5-HT [22]. These 5-HT activated cytokines and chemokine are produced by local neutrophils, monocytes/macrophages, dendritic cells, and T cells [51–53] and are known to induce MMP-3 expression [37, 38]. MMP-9 is predominantly synthesized in inflammatory cells, and the consequence of MMP-9 induction draws more leukocytes from the circulation to colon mucosa to worsen inflammation [54]. Furthermore, MMP-9 can potentiate IL-8 activity tenfold, further enhancing the chemokine activity [55]. Therefore, mice treated with combined 5-HT and low-dose DSS could produce as severe colitis as mice treated with high-dose DSS.

We noted that the expression of MMP-9 in gene and protein was no longer increased in the colons of mice treated with high-dose 5-HT in spite of having high levels of IL-6, IL-8, and TNF-*α* expression. It suggested that there is a dose-effect of 5-HT on MMP-9 expression. Apparently there is a negative feedback mechanism between MMP-9 protein and 5-HT biosynthesis, since in Mmp9^−/−^ mice, the TPH1 gene expression is highly regulated [56]. Therefore, the high level of 5-HT inhibits further induction of MMP-9 gene, although the exact mechanism is unknown.

While MMP-2 has the same function with that of MMP-9 in proteolytical degradation of gelatin, the expression of MMP-2 is ubiquitous and constitutive under physiological conditions [25, 26]. The expression of MMP-2 does not respond to inflammatory stimuli because MMP-2 gene lacks the binding site for inflammatory transcription factor [26]. It is possible that the high basal level of MMP-2 expression makes it more difficult to detect a low level of induction by 5-HT. This point is supported by the smaller fold of induction in DSS-treated colons; MMP-2 gene was induced 1.8- to 6.3-fold, whereas MMP-3 was induced 28- to 218-fold, and MMP-9 was 11- to 111-fold. Although we did not test the function of MMPs analyzed in this study, we suspect the elevated expression of MMP-9 aids, while MMP-2 suppresses inflammation as illustrated by several groups [57, 58]. The function of MMP-3 is less clear in the colon.

5-HT did not affect TIMP-1 and TIMP-2 gene expression, although DSS-induced strong inflammation induced both TIMP gene expressions. The similar pattern of induction of TIMPs and MMPs has been reported for IBD patients [59]. It was reported that the ratio of MMP-3/TIMP-1 is increased in inflamed colons compared with noninflamed colon tissues of IBD patients [60]. The results from human and animal model indicate that MMPs and TIMPs are involved in the process of inflammation and their expressions are regulated by multifactor and multistep, and cytokines and growth factors are involved in expression regulation of both MMPs and TIMPs [30, 61].

Judging from the higher fold of induction for TIMP-1 (10- to 115-fold) than TIMP-2 (3.8- to 6.0-fold) and the fold of induction of MMP-2 and MMP-9, it is consistent with the notion that MMP-2 is primarily inhibited by TIMP-2 while MMP-9 is mainly inhibited by TIMP-1 [62, 63]. In IBD patients after the remission of intestinal inflammation by Infliximab, a chimeric monoclonal anti-TNF-*α* antibody, the upregulated MMPs and TIMPs, including MMP-2, MMP-3, and MMP-9 and TIMP-1 and TIMP-2, were recessed [59]. Besides inhibition of MMPs, TIMPs have many other functions such as regulation of cell proliferation, cell migration, angiogenesis, and apoptosis [29].

## 5. Conclusion

Our findings show that 5-HT induces the expression of MMP-3 and MMP-9 in mouse colon. This is a direct effect since 5-HT induces MMP-3 mRNA levels in human colon cancer Caco-2 cells and MMP-9 mRNA in human primary culture of leucocytes. Our results support the notion that 5-HT exacerbates DSS-induced colitis by enhancing the production of MMP-3 and MMP-9. The significance of our findings is that we have provided a link between 5-HT-associated colitis and MMPs.

## Figures and Tables

**Table 1:** Primers sequence for qPCR.

Species	mRNA	Gene	Primer sequence		Amplicon (bp)
M	NM-031168.1	IL-6	Forward	5′-AACGATGATGCACTTGCAGA	128
			Reverse	5′-TGGTACTCCAGAAGACCAGAGG	
M	NM-011339.2	IL-8	Forward	5′-CTAGGCATCTTCGTCCGTCC	217
			Reverse	5′-TTGGGCCAACAGTAGCCTTC	
M	NM-013693.3	TNF-*α*	Forward	5′-CCACCACGCTCTTCTGTCTACT	161
			Reverse	5′-TGCTACGACGTGGGCTACA	
M	NM-008610	MMP-2	Forward	5′-ACAAGTGGTCCGCGTAAAGT	189
			Reverse	5′-GTAAACAAGGCTTCATGGGGG	
M	NM-010809.1	MMP-3	Forward	5′-AGGGATGATGATGCTGGTATG	210
			Reverse	5′-AACACCACACCTGGGCTTAT	
M	NM-013599	MMP-9	Forward	5′-GCCGACTTTTGTGGTCTTCC	80
			Reverse	5′-GGTACAAGTATGCCTCTGCCA	
M	NM-001044384	TIMP-1	Forward	5′-GACACACCAGAGCAGATACCAT	189
			Reverse	5′-TGGTCTCGTTGATTTCTGGGG	
M	NM-011594	TIMP-2	Forward	5′-GTGCAAGATCACTCGCTGTC	102
			Reverse	5′-TGGTGCCCATTGATGCTCTT	
M	NM-007393	*β*-Actin	Forward	5′-GGCTGTATTCCCCTCCATCG	154
			Reverse	5′-CCAGTTGGTAACAATGCCATGT	
H	NM-002422.3	MMP-3	Forward	5′-AGGGATGATGATGCTGGTATG	
			Reverse	5′-AACACCACACCTGGGCTTAT	174
H	NM-004994.2	MMP-9	Forward	5′-TTCAGGGAGACGCCCATTTC	
			Reverse	5′-TGGGTGTAGAGTCTCTCGCT	244
H	NM-001101	*β*-Actin	Forward	5′-CTCTTCCAGCCTTCCTTCCT	
			Reverse	5′-AGCACTGTGTTGGCGTACAG	116

M: mouse, H: human.

**Table 2:** The changes of body weight colon length and histopathology of mice in each group.

Group	Control	1 mg 5-HT	2 mg 5-HT	1% DSS	1% DSS + 1 mg 5-HT	2.5% DSS
*n*	8	9	9	14	12	7
Change of BW (%)^¶^	2.1 ± 2.4	1.8 ± 1.8	0.9 ± 3.3	−5.9 ± 4.2^*∗*^	−19.0 ± 5.9^*∗*^	−21.6 ± 5.9^*∗*^
Colon length (cm)	7.8 ± 0.5	7.6 ± 0.3	7.4 ± 0.4	6.4 ± 0.6^*∗*^	5.2 ± 0.7^*∗*^	4.9 ± 0.5^*∗*^
Colon H&E staining scores	0.1 ± 0.1	0.3 ± 0.1	0.4 ± 0.2	6.3 ± 0.9^*∗*^	10.7 ± 2.5^*∗*^	11.6 ± 1.9^*∗*^

^¶^Change of BW presents the change of mouse body weight at day 6.

^*∗*^ *P* < 0.05 versus control mice.

**Table 3:** 5-HT increases cytokine gene expression in mouse colon.

Group	Control	1 mg 5-HT	2 mg 5-HT	1% DSS	1% DSS + 1 mg 5-HT	2.5% DSS
*n*	8	9	9	14	12	7
IL-6	1 ± 0.1	1.4 ± 0.3	2.6 ± 0.6^*∗*^	144 ± 24.2^*∗*^	1722 ± 415.9^*∗*^	1070 ± 445.1^*∗*^
IL-8	1 ± 0.4	1.3 ± 0.3	3.7 ± 0.6^*∗*^	0.9 ± 0.1	1.8 ± 0.05	1.1 ± 0.1
TNF-*α*	1 ± 0.2	1.5 ± 0.1	1.7 ± 0.3	5.6 ± 0.8^*∗*^	8.5 ± 1.6^*∗*^	9.8 ± 1.2^*∗*^

^*∗*^ *P* < 0.05 versus control mice.

The data stands for relative fold of cytokine mRNA.

**Table 4:** Analysis of MMPs mRNA in mouse colon.

Group	Control	1% DSS	1% DSS + 1 mg 5-HT	2.5% DSS
MMP-2	1.0 ± 0.04	1.8 ± 0.2^*∗*^	2.9 ± 0.2^*∗*^	6.3 ± 0.3^*∗*^
MMP-3	1.0 ± 0.05	28.1 ± 12.2^*∗*^	218 ± 101.5^*∗*^	170 ± 37^*∗*^
MMP-9	1.0 ± 0.06	11.8 ± 2.0^*∗*^	52.7 ± 9.6^*∗*^	111.4 ± 19.8^*∗*^
TIMP-1	1.0 ± 0.03	9.8 ± 3.5^*∗*^	114.6 ± 27.3^*∗*^	82.1 ± 10.7^*∗*^
TIMP-2	1.0 ± 0.03	1.2 ± 0.2	6.0 ± 1.9^*∗*^	3.8 ± 0.5^*∗*^

^*∗*^ *P* < 0.05 versus control mice.

The data presents 8 mice of each group and stands for relative fold.

**Table 5:** Analysis of MMP-3 and MMP-9 protein levels by IHC in mouse colon.

Group	*n*	Control	1 mg 5-HT	2 mg 5-HT	1% DSS	1% DSS + 1 mg 5-HT	2.5% DSS
MMP-3	6	0.5 ± 0.5	0.8 ± 1.0	3.7 ± 0.8^*∗*§^	5.7 ± 2.0^*∗*§^	8.8 ± 2.0^*∗*§^	8.2 ± 3.8^*∗*§^
MMP-9	6	0.3 ± 0.5	3.0 ± 1.1^*∗*§^	0.7 ± 0.8	4.2 ± 1.0^*∗*§^	9.0 ± 2.7^*∗*§^	7.5 ± 4.2^*∗*§^

^*∗*^ *P* < 0.05 versus control mice; ^§^ *P* < 0.05 versus 1 mg 5-HT group for MMP-3 or versus 2 mg 5-HT group for MMP-9.
